# Diagnosis and management of Spigelian hernia: A review of literature and our experience

**DOI:** 10.4103/0972-9941.45204

**Published:** 2008

**Authors:** T Mittal, V Kumar, R Khullar, A Sharma, V Soni, M Baijal, P K Chowbey

**Affiliations:** Minimal Access and Bariatric Surgery Centre, Sir Ganga Ram Hospital, Old Rajinder Nagar, New Delhi – 110 060, India

**Keywords:** Extraperitoneal space, laparoscopy, spigelian hernia, total extraperitoneal approach, transperitoneal approach

## Abstract

Spigelian hernia occurs through slit like defect in the anterior abdominal wall adjacent to the semilunar line. Most of spigelian hernias occur in the lower abdomen where the posterior sheath is deficient. The hernia ring is a well-defined defect in the transverses aponeurosis. The hernial sac, surrounded by extraperitoneal fatty tissue, is often interparietal passing through the transversus and the internal oblique aponeuroses and then spreading out beneath the intact aponeurosis of the external oblique. Spigelian hernia is in itself very rare and more over it is difficult to diagnose clinically. It has been estimated that it constitutes 0.12% of abdominal wall hernias. The spigelian hernia has been repaired by both conventional and laparoscopic approach. Laparoscopic management of spigelian hernia is well established. Most of the authors have managed it by transperitoneal approach either by placing the mesh in intraperitoneal position or by raising the peritoneal flap and placing the mesh in extraperitoneal space. There have also been case reports of management of spigelian hernia by total extraperitoneal approach. We retrospectively reviewed our experience of ten patients between 1997 and 2007. Eight patients (8/10) presented with abdominal pain and two patients (2/10) were asymptomatic. In six patients (6/10) we performed an intraperitoneal onlay IPOM repair, in two patients (2/10) transabdominal preperitoneal repair (TAPP), and in two (2/10) total extraperitoneal repair (TEP). There were no recurrences, or other morbidity at mean follow up period of 3.2 years (range 6 months to 10 years).

## INTRODUCTION

Spigelian hernias occurs through slit like defects in the anterior abdominal wall adjacent to the semilunar line which extends from the tip of the ninth costal cartilage to the pubic spine at the lateral edge of the rectus muscle inferiorly. Most of spigelian hernias occur in the lower abdomen where the posterior sheath is deficient. It is also called “spontaneous lateral ventral hernia” or “hernia of semilunar line”. The hernia ring is a well-defined defect in the transversus aponeurosis. The diagnosis of spigelian hernia is difficult. The hernia may be interparietal with no obvious mass on inspection or palpation. The spigelian hernia has been repaired by both conventional and laparoscopic approaches. Most of the time when laparoscopy has been used as a treatment modality for spigelian hernia it has been done by transabdominal approach.[1–4] Total extraperitoneal repair (TEP) of spigelian hernia has also been reported in literature.[5,6] The advantage of TEP approach is that it eliminates the complications related to violation of peritoneal layer to reach the preperitoneal space.

## CLINICAL PRESENTATION

Symptoms can vary from abdominal pain, lump in the anterior abdominal wall or patient may have history of incarceration with or without intestinal obstruction. Pain varies in type, severity, and location and depends upon contents of hernia. Pain often can be provoked or aggravated by maneuvers that increase the intra abdominal pressure and is relieved by rest.

If patient has a palpable lump along the spigelian aponeurosis, the diagnosis is apparent. The same applies if the hernia appears when the patient is upright and disappears spontaneously on lying down. The clinical diagnosis of hernia is complicated by that the defect continues to expand laterally and caudally between two oblique muscles. Some patients present with abdominal pain but no lump. For these patients radiological investigations are required for diagnosis. If after radiological investigation the diagnosis is uncertain, diagnostic laparoscopy may be performed.

## OUR EXPERIENCE

We retrospectively reviewed our experience of ten patients with spigelian hernia between 1997 and 2007. Eight patients (8/10) had abdominal pain and two (2/10) were asymptomatic. Out of eight patients with abdominal pain, six patients with spigelian hernia were diagnosed on clinical examination and two patients were diagnosed on radiological examination (Ultrasound, CT Scan). Two patients (2/10) presented with occult hernias which were asymptomatic. One patient presented with a concomitant primary umbilical hernia and the other patient with bilateral inguinal hernia.

## OPERATIVE TECHNIQUES

### Conventional approach

A transverse incision is sited over the protrusion. External oblique aponeurosis is incised in the direction of its fibers to expose the peritoneal sac. The most common sac content is omentum but intestine, appendix, gall bladder, stomach or ovary have been reported.[7] Most surgeons simply invert the sac alone. The hernial orifice can be closed with sutures or prosthetic patch placed either in pre-peritoneal space or above the fascia.

### Endoscopic approaches

Intraperitoneal onlay mesh repair (IPOM): Intraperitoneal access is performed using Veress needle or open technique. Once abdominal access is obtained, site of hernial orifice is readily identified and ports are placed at least 10 cm away from the hernial defect in the form of an arc of a circle whose center is the hernial defect. Contents are reduced from the sac and adhesiolysis is performed if required to obtain an overlap of 5 cm around the defect for a synthetic mesh. The mesh is fixed using a combination of transabdominal sutures and tacks (Autosuture, Tyco health care, US surgicals, CT, USA).

### Transabdominal preperitoneal repair

**Figure 1:**
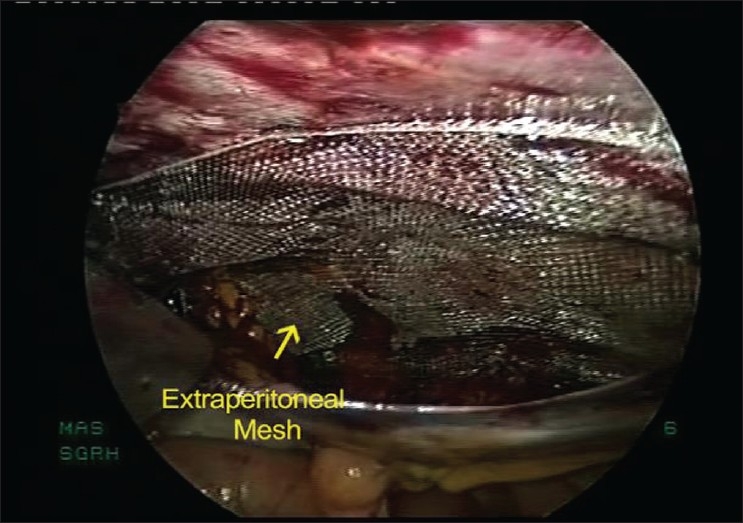
TAPP repair for spigelian hernia – after placement of mesh in extraperitoneal space

Once the hernia sac contents are reduced, a peritoneal flap is raised as in trans abdominal pre peritoneal (TAPP) approach and attempt is made to completely reduce the hernial sac. After dissecting the peritoneal flap for about 5 cm around the hernial defect, Prolene mesh (Ethicon, Inc., Somerville, NJ, USA) is placed in the dissected extraperitoneal space [Figure 1] and is fixed using tacks (Autosuture, Tyco health care, US surgicals, CT, USA). The peritoneal flap is closed either with tacks or with a continuous suture.

### Total extraperitoneal repair

**Figure 2:**
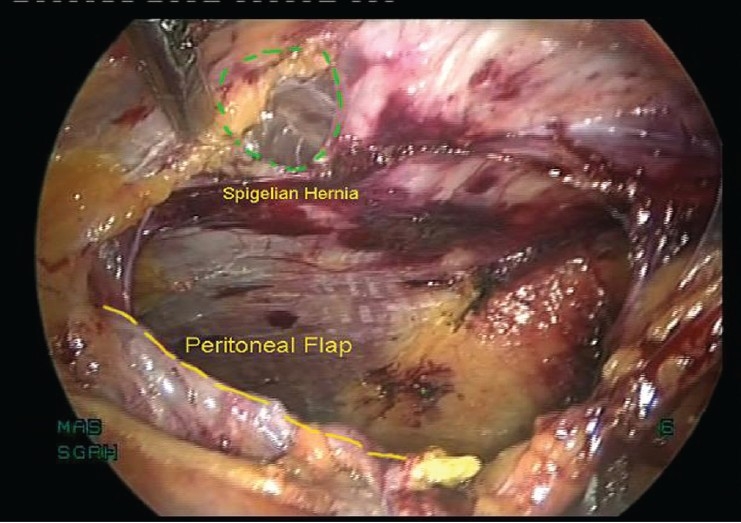
TEP repair for spigelian hernia – after reduction of hernia sac

Endoscopic TEP repair is performed using 3 midline ports. Extraperitoneal space is created by open access and a balloon. The spigelian hernial sac is identified around arcuate line and reduced completely [Figure 2]. The peritoneum is dissected above the arcuate line to have a 5 cm margin around the hernial defect for mesh overlap. A Prolene mesh (Ethicon, Inc., Somerville, NJ, USA) is used to cover the hernial defect. Mesh is fixed to anterior abdominal wall with spiral tacks (Autosuture, Tyco health care, US surgicals, CT, USA).

### Our experience

We retrospectively reviewed our experience of ten patients with spigelian hernia between 1997 and 2007. In six patients (6/10) we performed IPOM repair, in two patients (2/10) TAPP repair and in two (2/10) TEP repair. Two patients with occult spigelian hernias had concomitant IPOM repair for primary umbilical hernia and TEP repair for bilateral inguinal hernia. All our patients had an uneventful recovery. There were no recurrences, mesh infection or chronic pain with mean follow up period of 3.2 years (range 6 months to 10 years).

## DISCUSSION

Spigelian hernia is named after Adriaan van Spieghel, who described the semilunar line. However, the hernia was first described by Klinkosch in 1764.[8] The hernia appears to peak in the 4^th^ to 7^th^ decades. The male to female ratio is 1:1.18.[9] Spigelian hernias are very uncommon and constitute only 0.12% of all abdominal wall hernias.[7]

Spigelian hernia can be congenital or acquired.[10] Perforating vessels may weaken the area in spigelian fascia and a small lipoma or fat enters here which gradually leads to hernia formation. Spigelian hernia may be related to stretching in the abdominal wall caused by obesity, multiple pregnancies, previous surgery or scarring. Spigelian hernia has been described as a complication of chronic ambulatory peritoneal dialysis (CAPD).[11]

**Figure 3:**
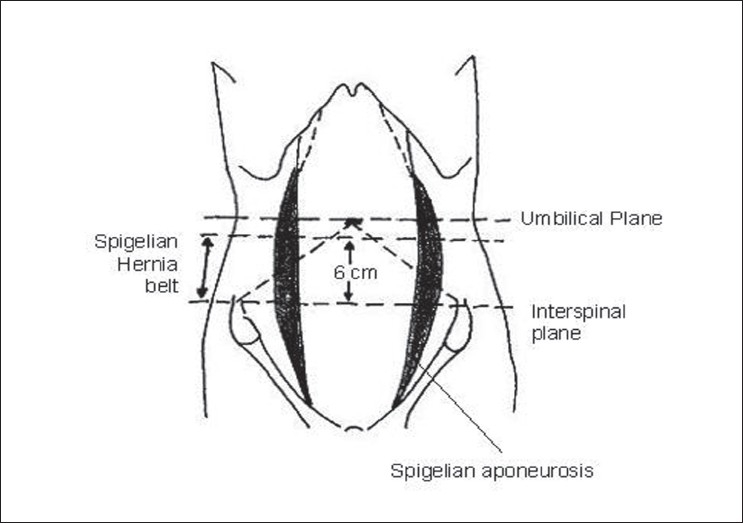
Site of spigelian hernia belt

The spigelian aponeurosis is widest between 0 and 6 cm cranial to the interspinous plane and 85-90% of the hernias occur within this “spigelian hernia” belt [Figure 3]. The hernial ring is a well-defined defect in the aponeurosis. The hernial sac, surrounded by extraperitoneal fat, is often interparietal passing through the transversus and the internal oblique aponeuroses and then spreading out beneath the intact aponeurosis of the external oblique, or lying in the rectus sheath alongside the rectus muscle.

The diagnosis of a spigelian hernia is difficult; few surgeons suspect it, it has no characteristic symptoms, and the hernia may be interparietal with no obvious mass on inspection or palpation. Only 50% of cases are diagnosed preoperatively.[12,13] It may present as a swelling adjacent to the iliac crest. The patient may have a classic lump when he/she stands up. The lump is painful if the patient stretches and disappears on lying down. Sometimes the local discomfort can be confused with peptic ulceration. Rarely the hernia can enter the rectus sheath and can be confused with spontaneous rupture of rectus muscle or with a hematoma in the rectus sheath.

Ultrasound is recommended as first line imaging investigation. With this aid, the correct diagnosis was obtained in 19 of 24 cases studied.[9] Ultrasonic scanning of the semilumar line should be under taken in all patients with obscure abdominal pain associated with bulging of the belly wall in the standing patient. The advantages of real time ultrasonography is the ability to perform examination in both supine and upright positions and while patient performs a Valsalva maneuver. Now CT scanning with close thin sections is considered the most reliable technique to make the diagnosis in doubtful cases.[14] The use of oral contrast medium during the examination is recommended so that any bowel content can be identified. The increasing availability of the magnetic resonance imaging (MRI) may be of benefit in the preoperative evaluation of these difficult cases.

The differential diagnosis includes appendicitis and appendiceal abscess, a tumor of the abdominal wall or a spontaneous hematoma of the rectus sheath or even acute diverticulitis.[15]

Spigelian hernias are treacherous and have a real risk of strangulation. The risk of strangulation is higher because of sharp fascial margin around the defect. Richter type of hernia has also been reported to occur with spigelian hernia. For this reason, surgery should be advised in all patients.

Surgery can be performed either by open technique or by laparoscopically. Carter and Mizes performed first intraabdominal laparoscopic repair of spigelian hernia in 1992.[1] They used sutures to close the defect. After that there have been multiple reports of successful management of spigelian hernia by laparoscopy.[1–4] In these reports, mesh is placed either intraperitoneally or extraperitoneally after creating a peritoneal flap by trans abdominal approach. In the only prospective randomized controlled trial comparing conventional versus laparoscopic management of spigelian hernia (11 conventional and 11 laparoscopically) there was significant advantage in terms of morbidity and hospital stay in laparoscopy group.[16]

There have also been case reports of management of spigelian hernia by total extraperitoneal approach.[5,6] The advantage of extraperitoneal placement of mesh is that Prolene mesh can be used, which decreases the cost of procedure, also, incidence of complications like intestinal obstruction and fistulization of bowel is expected to decrease (which can occur with intraperitoneal placement of mesh). As compared to transabdominal extraperitoneal approach, the TEP approach eliminates the complications related to violation of peritoneal layer to reach the preperitoneal space. The need to close the peritoneal flap with tacks or sutures (in TAPP approach) also increases the operative time and cost.

## CONCLUSION

Spigelian hernias are clinically elusive often until strangulation occurs. If diagnosed, operation should always be advised. These can be repaired successfully by laparoscopic method (intraperitoneal or extraperitoneal approach) to confer all advantages of laparoscopy to patients.
